# Case series: Maraviroc and pravastatin as a therapeutic option to treat long COVID/Post-acute sequelae of COVID (PASC)

**DOI:** 10.3389/fmed.2023.1122529

**Published:** 2023-02-08

**Authors:** Bruce K. Patterson, Ram Yogendra, Jose Guevara-Coto, Rodrigo A. Mora-Rodriguez, Eric Osgood, John Bream, Purvi Parikh, Mark Kreimer, Devon Jeffers, Cedric Rutland, Gary Kaplan, Michael Zgoda

**Affiliations:** ^1^IncellDX Inc., San Carlos, CA, United States; ^2^Department of Anesthesiology, Beth Israel Lahey Health, Burlington, MA, United States; ^3^Centro de Investigación en Cirugía y Cáncer (CICICA), Universidad de Costa Rica, San Jose, Costa Rica; ^4^Lab of Tumor Chemosensitivity, CIET/DC Lab, Faculty of Microbiology, Universidad de Costa Rica, San Jose, Costa Rica; ^5^Department of Medicine, St. Francis Medical Center, Trenton, NJ, United States; ^6^Department of Emergency Medicine, Novant Health Kernersville Medical Center, Kernersville, NC, United States; ^7^Department of Allergy and Immunology, NYU Langone Tisch Hospital, New York, NY, United States; ^8^Department of Emergency Medicine, New York Presbyterian Hospital, Brooklyn, NY, United States; ^9^Department of Anesthesiology, Stamford Hospital, Stamford, CT, United States; ^10^Rutland Medical Group, Newport Beach, CA, United States; ^11^Department of Community and Family Medicine, Georgetown University Medical Center, Washington, DC, United States; ^12^Department of Medicine, Creighton University School of Medicine, Phoenix, AZ, United States

**Keywords:** long COVID, maraviroc, CCR5 antagonist, PASC, statins, fractalkine (CX3CR1)

## Abstract

Post-acute sequelae of COVID (PASC), or long COVID, is a multisystem complication of SARS-CoV-2 infection that continues to debilitate millions worldwide thus highlighting the public health importance of identifying effective therapeutics to alleviate this illness. One explanation behind PASC may be attributed to the recent discovery of persistent S1 protein subunit of SARS-CoV-2 in CD16+ monocytes up to 15 months after infection. CD16+ monocytes, which express both CCR5 and fractalkine receptors (CX3CR1), play a role in vascular homeostasis and endothelial immune surveillance. We propose targeting these receptors using the CCR5 antagonist, maraviroc, along with pravastatin, a fractalkine inhibitor, could disrupt the monocytic-endothelial-platelet axis that may be central to the etiology of PASC. Using five validated clinical scales (NYHA, MRC Dyspnea, COMPASS-31, modified Rankin, and Fatigue Severity Score) to measure 18 participants’ response to treatment, we observed significant clinical improvement in 6 to 12 weeks on a combination of maraviroc 300 mg per oral twice a day and pravastatin 10 mg per oral daily. Subjective neurological, autonomic, respiratory, cardiac and fatigue symptoms scores all decreased which correlated with statistically significant decreases in vascular markers sCD40L and VEGF. These findings suggest that by interrupting the monocytic-endothelial-platelet axis, maraviroc and pravastatin may restore the immune dysregulation observed in PASC and could be potential therapeutic options. This sets the framework for a future double-blinded, placebo-controlled randomized trial to further investigate the drug efficacy of maraviroc and pravastatin in treating PASC.

## Introduction

Post-acute sequelae of COVID (PASC), commonly referred to as long COVID, is an emerging public health syndrome that continues to devastate and debilitate adult and pediatric survivors of acute SARS-CoV-2 infection. The World Health Organization (WHO)-led Delphi consensus defined PASC as a syndrome starting 3 months from onset of probable infection with symptoms lasting over 2 months and could not be explained by an alternative diagnosis (1). Over 200 symptoms have been attributed to PASC (2) thus posing an enormous challenge clinically. The multi-organ involvement causes cognitive impairment, debilitating neuropathy, chronic migraines, autonomic dysfunction, cardiac dysrhythmias, dyspnea at rest, severe fatigue, and myalgias (3). Presently, minimal therapeutic options are available to treat PASC which can be attributed to the pathology not yet being fully described. However, we recently reported that the S1 protein subunit of SARS-CoV2 is retained in both non-classical (CD14− CD16+) and intermediate (CD14+ CD16+) monocytes several months after acute infection. Typically, these monocytes persist only for a few days, but in PASC patients, the S1 containing monocytes can persist for months and years (4), which we propose contributes to the pathophysiology behind PASC. Non-classical monocytes are involved in phagocytosis and vascular adhesion by patrolling the endothelium under homeostatic and inflammatory conditions through B2 integrin, lymphocyte function-associated antigen-1 (LFA-1) and high levels of fractalkine receptors (CX3CR1) (5, 6). On the other hand, CD14+ CD16+ monocytes express high levels of C-C chemokine receptor type 5 (CCR5) and fractalkine receptors and are involved in antigen presentation, cytokine secretion and apoptosis regulation (6, 7). Since CCR5 and fractalkine receptors have been studied for various chronic inflammatory pathologies, we hypothesized that both these receptors may also be therapeutic targets for PASC. CD16+ monocytes also produce high levels of various pro-inflammatory cytokines which could be an explanation for the heterogenous symptomatology in PASC. Specifically, elevations in C-C chemokine ligand 5 (CCL5)/RANTES (Regulated on Normal T-cell Expression and Secretion), IL-2, IL-6, IFN-gamma and Vascular Endothelial Growth Factor (VEGF), along with decrease in CCL4 have been observed in patients and are hypothesized to be contributing to the pathophysiology of PASC (8).

Here, we describe an 18 participant case series investigating the combination of the CCR5 receptor antagonist maraviroc, and pravastatin, which targets fractalkine, as a potential therapeutic approach in addressing and treating the potential pathology of PASC. The CCR5 receptor is a seven-transmembrane G protein-coupled receptor (GPCR) that is found on macrophages and T-lymphocytes and functions to regulate trafficking and effector functions of these cells (9). The role of CCR5 as a co-receptor for human immunodeficiency virus (HIV) entry was discovered in 1996. Maraviroc is the first and only US Food and Drug Administration (FDA) and European Medical Agency (EMA) approved CCR5 receptor antagonist available to date. Maraviroc is a negative allosteric modulator of the CCR5 receptor, and by binding to the CCR5 receptor, it induces receptor conformational changes that prevent the chemokine binding of RANTES (CCL5) and CCR5-mediated signaling (10). While this mechanism has been researched and studied extensively in HIV infection, there is increasingly greater recognition and appreciation of the CCR5-CCL5 axis in many other conditions and pathologies such as cancer, autoimmune disorders and endothelial dysfunction. This signaling is central to the pathophysiology of inflammation by directing immune cells through a process called chemotaxis. These actions are mediated through RANTES, which is produced by platelets, macrophages, eosinophils, fibroblasts, endothelial, epithelial and endometrial cells (11). The effects of RANTES have been implicated in respiratory tract infections, especially viruses possessing RNA genome (including coronavirus, influenza, RSV, and adenovirus), asthma, neuroinflammation, and atherosclerosis (12, 13). Maraviroc has also been documented to restore the homeostasis of regulatory T-cells (Treg), increase CD4 and CD8 positive counts, and inhibit HIV-associated chronic inflammation and activation (14, 15). Interestingly, CD4 and CD8 positive T-cells expressing PD-1 and T-regs have been observed to be significantly lower in PASC patients compared to healthy controls (8), thus suggesting maraviroc could restore the immune dysregulation seen in PASC. The commonly known mechanism of action of statins is inhibition of hydroxymethylglutaryl-CoA (HMG-CoA) reductase enzyme in lowering cholesterol. However, statins have also been implicated in reducing inflammation, suppressing fractalkine, and lowering VEGF and IL-6 (16), and as such, may play a role in the pathophysiology of PASC. We targeted fractalkine using pravastatin since CD16+ monocytes express high levels of the fractalkine receptor believing this may address the elevations in vascular markers seen in PASC.

## Materials and methods

The medical records and immunological lab reports from 18 adult PASC patients treated with maraviroc 300 mg per oral twice daily and pravastatin 10 mg per oral daily by independent private practice physicians and clinics were collected and analyzed. The CCTC is a virtual consultation group that works in collaboration with these physicians to collect and analyze immunological data. The 18 patients selected for this case series were from a pool of patients who reported symptom improvement while on maraviroc and pravastatin and who fit the inclusion and exclusion criteria we set below:

5 patients were previously treated with ivermectin, 2 with fluvoxamine, and 1 with prednisone.

### Inclusion criteria

All the participants in the case series were COVID-19 survivors with documented FDA EUA approved RT-PCR SARS-CoV2 positive test and/or were positive for anti-SARS-CoV2 antibodies using FDA EUA approved tests. All participants had one or more new onset symptoms that persisted greater than 3 months after the diagnosis of acute COVID-19 infection. These symptoms included cognitive impairment (brain fog), migraines, post exertional malaise (PEM), myalgias, arthralgias, severe fatigue, tachyarrhythmias, postural orthostatic tachycardia syndrome (POTS), and shortness of breath. All participants displayed either isolated or combinations of elevated pro-inflammatory markers: RANTES, TNF-alpha, IFN-gamma, sCD40L, VEGF, IL-6, IL-2, and IL-8 on the IncellKINE panel. The IncellKINE cytokine panel is a set of 14 cytokines that was constructed from a previous machine-based learning algorithm that identified potential markers of PASC (8).

### Exclusion criteria

We excluded participants with a pre-COVID history of migraines, neuropathy, inflammatory bowel disease, depression and anxiety disorders, chronic fatigue syndrome, Epstein-Barr infection, Lyme disease, fibromyalgia, arthritis, COPD, asthma, chronic kidney disease, chronic heart failure (CHF), arrhythmias, bleeding disorders, and anticoagulation therapy.

### Treatment endpoints

When patients reported that their post-COVID symptoms improved by 80% of greater, we initiated discontinuation of both maraviroc and pravastatin. Bi-monthly follow-ups were conducted for 12 weeks post-treatment to monitor if symptoms reappeared or worsened.

### Validated scoring system for patient assessment before and after treatment

A challenge in studying and defining PASC is the heterogenous clinical presentation and multisystem involvement. Thus, we categorized the main participant symptoms into 5 groups: neurological/autonomic function, cardiac, respiratory, overall functionality, and fatigue. Since there are no validated scales for PASC, we used five validated scales for other organ systems [New York Heart Association (NYHA), Modified Rankin Scale for Neurologic Disability, Fatigue Severity Scale (FSS), COMPASS-31 and Medical Research Council (MRC) Dyspnea Scale, respectively] to measure subjective participant responses to treatment (Tables 1–5). Participants were administered validated self-questionnaires about their PASC symptoms before and after treatment with maraviroc and pravastatin treatment. The length of duration of treatment varied based on repeat immune markers and participant-reported symptom improvement. Since many of these participants were on other medications and anti-inflammatories prior to starting maraviroc and pravastatin, the biomarkers and subjective data presented are from the onset of this combination. Phone interviews were conducted with each participant before and after subjective responses to the medications.

**TABLE 1:** Patient information and demographics.

	Sex	Age	Months from positive COVID test to testing	Weeks on maraviroc and pravastatin	Hospitalized Y/N
P1	F	50	9	10	N
P2	M	59	10	8	N
P3	F	43	8	6	N
P4	F	27	7	8	N
P5	F	33	5	8	N
P6	M	30	14	6	N
P7	F	46	14	8	N
P8	M	50	10	6	N
P9	F	53	4	6	N
P10	F	53	14	12	N
P11	M	57	17	6	N
P12	M	47	9	10	Y
P13	F	46	6	8	N
P14	F	40	8	8	N
P15	M	45	17	8	N
P16	M	63	10	7	N
P17	M	18	8	6	N
P18	F	43	16	12	N

**TABLE 2:** New York Heart Association (NYHA) functional classification.

**Class 1**	No limitation of physical activity. Ordinary physical activity does not cause undue fatigue, palpitation, dyspnea (shortness of breath).	**Class 2**	Slight limitation of physical activity. Comfortable at rest. Ordinary physical activity results in fatigue, palpitation, dyspnea (shortness of breath).	**Class 3**	Marked limitation of physical activity. Comfortable at rest. Less than ordinary activity causes fatigue, palpitation, or dyspnea.	**Class 4**	Unable to carry on any physical activity without discomfort. Symptoms of heart failure at rest. If any physical activity is undertaken, discomfort increases.

**TABLE 3:** Medical Research Council (MRC) dyspnea scale.

**Grade 1**	Are you ever troubled by breathlessness except on strenuous exertion?	**Grade 2**	Are you short of breath when hurrying on the level or walking up a slight hill?	**Grade 3**	Do you have to walk slower than most people on the level? Do you have to stop after a mile or so (or after 15 min) on the level at your own pace?	**Grade 4**	Do you have to stop for breath after walking about 100 yds. (or after a few minutes) on the level?	**Grade 5**	Are you too breathless to leave the house, or breathless after undressing?

**TABLE 4:** Modified Rankin scale.

**0**	No symptoms	**1**	No significant disability despite symptoms; able to carry out all usual duties and activities	**2**	Slight disability; unable to carry out all previous activities, but able to look after own affairs without assistance	**3**	Moderate disability; requiring some help, but able to walk without assistance	**4**	Moderately severe disability; unable to walk without assistance and unable to attend to own bodily needs without assistance	**5**	Severe disability; bedridden, incontinent and requiring constant nursing care and attention

**TABLE 5:** Fatigue Severity scale.

1	My motivation is lower when I am fatigued.	2	Exercise brings on excessive fatigue.	3	I am easily fatigued.	4	Fatigue interferes with my physical functioning.	5	Fatigue causes frequent problems for me.	6	My fatigue prevents sustained physical functioning.
7	Fatigue interferes with carrying out certain duties and responsibilities.	8	Fatigue is among my three most disabling symptoms.	9	Fatigue interferes with my work, family, or social life.						

The **New York Heart Association (NYHA) functional classification** was used to classify severity of PASC associated cardiac symptoms.

The **Composite Autonomic Symptom Scale 31 (COMPASS 31)**, a self-rating questionnaire consisting of 31 items and evaluating orthostatic intolerance, vasomotor, secretomotor, gastrointestinal, bladder, and pupillomotor function, was used to measure autonomic dysfunction and the subsequent therapeutic effects of maraviroc and pravastatin. A sub raw score for each of the six domains was calculated and converted into a weighted sub-score. The sum of this weighted sub-score gave a total score which ranged from 0 to 100, with 0 meaning no autonomic symptoms and 100 reflecting the most severe autonomic symptoms.

**Medical Research Council (MRC) Dyspnea scale** is a validated method comprised of five statements that aims to measure perceived feeling of breathlessness.

The **Modified Rankin scale for neurologic disability** is a validated scale to measure degree of disability after suffering a stroke or neurological insult.

The **Fatigue Severity Scale (FSS) questionnaire** is a nine-statement validated scale that rates the severity of fatigue symptoms. Participants were asked how accurately each statement reflected their condition before and after treatment with maraviroc and pravastatin and the extent to which they agreed or disagreed based on a scale of 1 (strongly disagree) to 7 (strongly agree).

### Serum cytokine measurements from participants: Multiplex cytokine quantification

Fresh plasma was used for cytokine quantification using a customized 14-plex bead based flow cytometric assay (IncellKINE, IncellDx, Inc., San Carlos, CA, United States) on a CytoFlex flow cytometer as previously described (8) using the following analytes: “TNF-α,” “IL-4,” “IL-13,” “IL-2,” “GM-CSF,” “sCD40L,” “CCL5 (RANTES),” “CCL3 (MIP-1α),” “IL-6,” “IL-10,” “IFN-γ,” “VEGF,” “IL-8,” and “CCL4 (MIP-1β)”. For each participant sample, 25 μL of plasma was used in each well of a 96-well plate.

End points for the study were when the patients reported improvement in post-COVID symptoms that led to increases in daily functionality and quality of life. We checked in with the patients every 2 weeks to monitor progress.

### Data acquisition and preprocessing

In total there were 18 unique individuals, with each individual being represented in duplicate for before and after treatment. The presence of a pre and post treatment for each individual categorized as PASC allowed us the possibility to separate the data set into a before and after data sets for the required statistical comparisons. To separate the before and after groups, we used the *python* programming language (version 3.9) and the *pandas* library (17, 18), which allowed us to group the samples according to before and after treatment. Once we separated the data in the two data sets, we then conducted the necessary comparative statistical analysis, including the statistical test to determine if there were significant differences between the two groups.

### Wilcoxon’s paired test to compare the before and after treatment groups

To determine if there were differences between the biomarker’s levels of the two groups (before and after) we compared the datasets by implementing the non-parametric Wilcoxon’s paired test. The implementation of this test was done using the python library *scipy* (19). The selection of the Wilcoxon test was based on the assumption that this non-parametric test does not assume normal distribution of the variables. Additionally, in contrast to parametric tests like ANOVA, Wilcoxon’s paired test does not base its comparison on the mean but median values. For our data we compared group *before* and group *after* with two alternative hypotheses. The first was a two-sided test, which resulted in a *p*-value less than 0.05. Subsequently, we tested for an alternative hypothesis “*greater*,” resulting in a *p*-value of less than 0.05.

### Correlation analysis between biomarker levels and subjective scores

In order to identify potential statistically significant relationships between the biomarkers present in the dataset and the subjective scores, we imported the full dataset into the R programming language (version 4.1.1) (20) and conducted a correlation analysis. The correlation analysis was calculated using the Pearson correlation coefficient, which allows the measurement of both strength and direction of the linear relationship between two variables.

The Pearson correlation coefficient has the advantage that its values are highly interpretable, always ranging from −1 (strong negative correlation) to +1 (strong positive correlation). Correlation coefficients were calculated for both the before and after data points, and to validate their statistical significance, their *p*-value was calculated. We defined that correlation coefficients were statistically significant if their *p*-value was equal or less than 0.05. In order to properly interpret and convey the correlation relationships and their statistical significances, we constructed a modified correlation matrix using a dot plot with the python programming language. We implemented additional functions and arguments that allowed us create a diagram only showing statistically significant correlations. Additionally, because correlation plot is constructed using a matrix, there is a mirror-image effect between the left and right side of the diagonal. We used the arguments of the plot to avoid this effect, in order to improve plot interpretability.

The dot plot implemented a color and size code. In this system, positive correlation coefficient were associated with shades of blue, whereas negative correlation values where associated with red correlation values. Regarding the size of the dot, it represented statistical significance, with greater sizes being indicative of greater statistical significance, smaller dots representing lesser values of significance, and no dots indicating no significance.

### Validation of long hauler status using a machine learning classifier

To determine the state of individuals in the dataset as Post-acute sequelae SARS-CoV-2 (PASC) we classified them using a machine learning model. This model, which consisted of a random forest classifier, developed in a previous report (8) and used to determine if samples belonged to disease state (Normal/Control, Mild-Moderate, Severe or PASC), was given the dataset as a prediction set. The implementation of this model was to confirm the disease status of individuals in the dataset as PASC. The random forest classifier was trained using a dataset consisting of 224 instances representing Normal/Control, Mild-Moderate, Severe or PASC. The classes in the dataset were imbalanced, and to address this, we implementing synthetic oversampling of the minority class (SMOTE) (21), which creates minority class instances by interpolation. The model using a balanced trainings set, was subsequently implemented in the unseen the dataset. The predictions of before and after individuals were outputted as an array, with a number indicating the predicted class.

## Results

### Comparison between “before” and “after” treatment demonstrates statistical differences between groups

The Wilcoxon paired test to contrast the before and after treatment groups using a two-sided hypothesis revealed the existence of statistically significant differences (*p*-value = 2.20e-17) between the cytokine profiles of the two treatment groups (before and after). Group 1 was defined as the before treatment and group 2 was after treatment. These results support that the medians of both groups are different and that a one-sided test was required to define the difference between group 1 and group 2. For the one-sided test, we focused on determining if the medians values for the biomarkers in treatment group 1 (before) were greater than those of group 2 (after). The results indicated that before treatment had statistically significant greater (*p*-value = 1.10e-17) magnitudes for the cytokines than the after-treatment group. The statistical analysis using a two-tailed and a one-tailed Wilcoxon test of the individuals from the maraviroc and pravastatin group indicated that there were statistically significant differences between the before and after treatment. The two-tailed test indicated that there were differences in the medians of these two groups (*p*-value = 2.20e-17). The one-sided test (*p*-value = 1.10e-17) indicated that before treatment had greater magnitudes for the cytokines (biomarkers) than the after treatment.

### Correlation analysis indicates the presence of positive correlations between cytokine biomarkers and subjective scores

**FIGURE 1:**
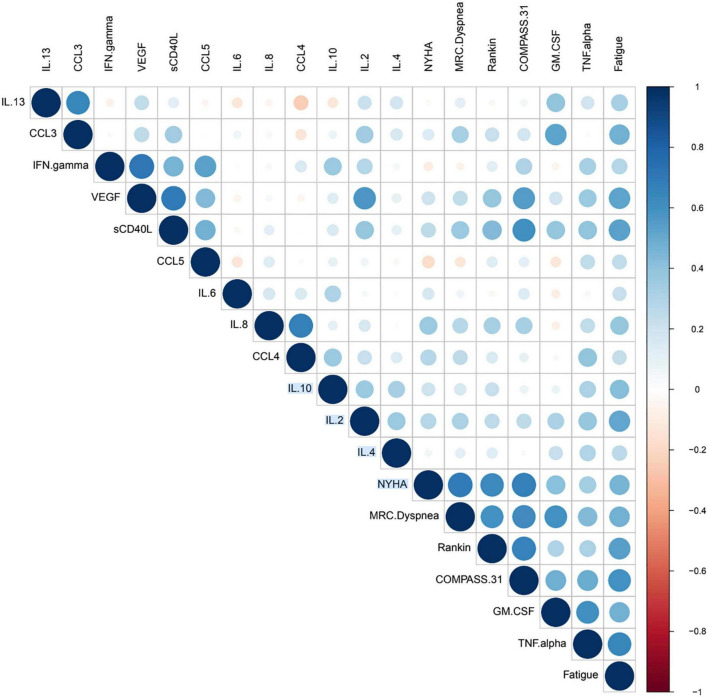
Correlation dot plot.

The correlation dot plot indicates the presence of significant correlations based on color and size of the dot (circle) on the right of the diagonal. The correlation value is indicative of the joint correlation for both the before and after treatment groups when compared to each variable. The diagonal correlation values are the values of each marker or subjective score compared against itself, and thus the coefficient will be equal to 1 (dark blue) and have the largest size possible dot. For the observed correlation coefficients, the intensity of the color was indicative of values of r (correlation coefficients) including various negligible positive and negative correlations, and instances ranging from low positive to moderate positive correlation, however we also identified the presence of negative correlation. Identification of correlation values was done by comparing with color scale in Figure 1, where dark blue indicated highly positive correlation (+1), and dark red indicated highly negative correlation (−1). The dot plot omitted non-significant correlations, which are represented as blank spaces. This permitted us to focus on the statistically significant relationships, and more specifically those between cytokines and subjective scores.

**FIGURE 2:**
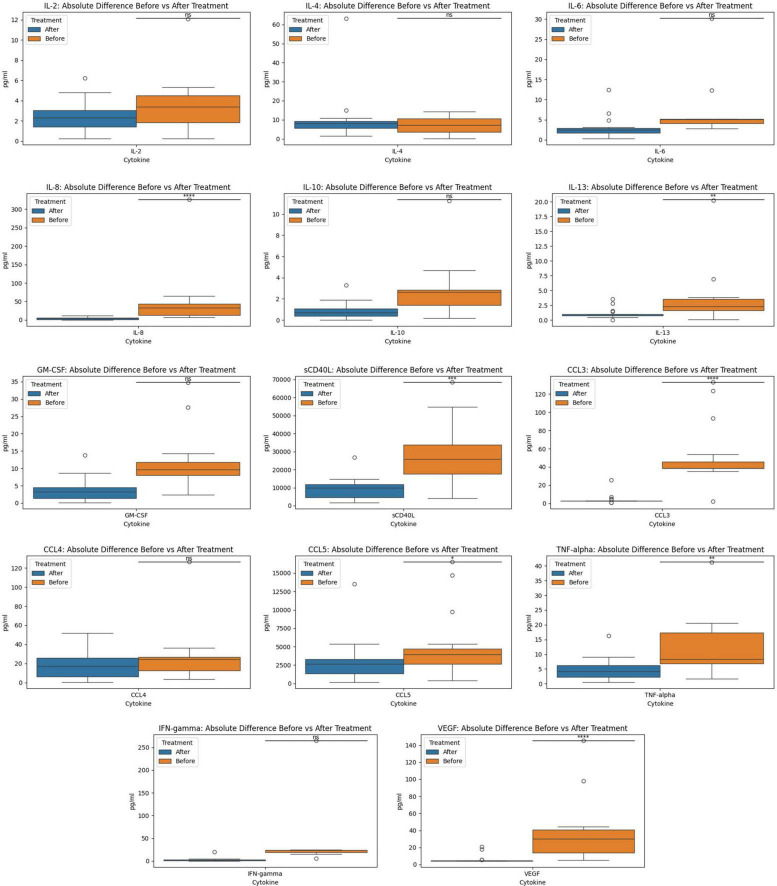
Before and after treatment individual cytokine measurement comparisons. The box plot represent the statistical comparison using the Wilcoxon paired test between the two treatment groups (before and after).

Additionally, it is possible to see that for the subjective scores (NYHA, MRC Dyspnea, Rankin, COMPASS 31 and Fatigue) there were multiple significant correlations with biomarkers. These significant correlations followed the trend of being mostly low positive or moderate positive, as indicated by the color of the dot (Figure 1). The presence of these cytokines and their significance values can be referenced in Figure 2 and Table 6, where positive significant correlations are reported. As indicated, we identified low negative correlation values, which were found for between CCL5 and both the subjective scores NYHA and MRC Dyspnea.

**TABLE 6:** Statistically significant positive Pearsons correlation coefficients between subjective scores and cytokines.

Score	Biomarker correlation		
Rankin	sCD40L	CC: 0.42	*p* = 0.01
	VEGF	CC: 0.40	*p* = 0.02
COMPASS-31	sCD40L	CC: 0.6	*p* = 0.0001
	VEGF	CC: 0.6	*p* = 0.0005
	GM = CSF	CC: 0.5	*p* = 0.002
	TNF-alpha	CC: 0.5	*p* = 0.0026
NYHA	IL-8	CC: 0.4	*p* = 0.03
	GM-CSF	CC: 0.4	*p* = 0.01
MRC Dyspnea	sCD40L	CC: 0.4	*p* = 0.04
	IL-2	CC: 0.4	*p* = 0.05
	TNF-alpha	CC: 0.4	*p* = 0.01
	GM-CSF	CC: 0.6	*p* = 0.0002
Fatigue Severity	sCD40L	CC: 0.5	*p* = 0.001
	IL-2	CC: 0.6	*p* = 0.0005
	TNF-alpha	CC: 0.7	*p* = 4 × 10^–5^
	VEGF	CC: 0.5	*p* = 0.001
	GM-CSF	CC: 0.5	*p* = 0.004

We analyzed the linear relationship between the cytokine biomarkers and the modified Rankin score (22). In brief, this is a 6-point disability scale that ranges from 0 (individual has no residual symptoms) to 5 (the individual is bedridden, incontinent and requires continuous care). According to the documentation an additional value of 6 is included for deceased or “expired” individuals. For the Rankin subjective score, we identified a low positive correlation with statistical significance for two biomarkers, VEGF and sCD40L (Table 6). Finally, we did the correlation analysis for the COMPASS 31 score (23). This scale was developed as a robust statistical instrument to determine autonomic symptoms, thus providing relevant severity scores for clinical assessment. For this scale, we identified that several cytokines had statistically significant relationships to the subjective score (Figure 1). TNF-alpha and GM CSF had low positive correlations, while VEGF and sCD40L showed moderate positive correlation (Table 6).

**FIGURE 3:**
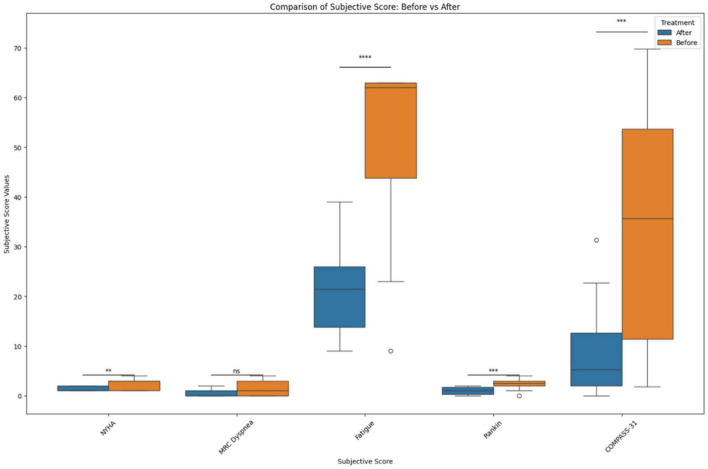
Before and after treatment individual subjective score comparisons. The box plot represents the statistical comparison using the Wilcoxon paired test between the two treatment groups (before and after). Statistical significance intervals are represented with asterisks (*), where ns indicates non-significant. *1.00e–02 < *p* ≤ 5.00e–02, **1.00e–03 < *p* ≤ 1.00e–02, ***1.00e–04 < *p* ≤ 1.00e–03, and ****p ≤ 1.00e–04.

Applying the New York Heart Association (NYHA) Functional Classification, which labels individuals in one of four categories, we were able to identify two statistically significant biomarkers in the joint correlation (Figure 3 and Table 6). The cytokines IL-8 and GM-CSF showed a low positive correlation to the NYHA score, with both having *r* values between 0.30 and 0.50. The linear association between IL-8 and GM-CSF indicates that there appears to be a weak linear association between both treatment groups (before and after) where the levels of both cytokines appear to be positively associated with the NYHA score. When subsequently analyzed the correlation values for the Medical Research Council (MRC) Dyspnea scale score (Figure 3), which is a simple scale allowing participants to indicate the effects of breathlessness on mobility, we were able to identify that for both treatment groups (joint correlation), the biomarkers GM-CSF, TNF-alpha and sCD40L presented statistically correlations. In the case of GM-CSF, the linear association between the cytokine and the subjective score was 0.593, which makes it a moderate positive correlation. For TNF-alpha and sCD40L there correlation values were in ranges between 0.30 and 0.50, indicating their association with the MRC Dyspnea score were low positive.

In addition, the correlation analysis of the Fatigue score from the Shirley Ryan Ability Lab at the Rehabilitation Institute of Chicago^1^ provides a 9-item scale allowing the measurement of the effects of fatigue on an individual. The scores range from a value of 9 (lowest possible score) to 63 (highest fatigue effects). Our analysis identified that various biomarkers showed statistically significant correlations (Figure 3). These linear associations were present in both the before and after treatment groups (joint correlation). The cytokines IL-2, sCD40L, TNF alpha and VEGF presented a positive correlation, with *r* values ranging between 0.50 and 0.70, as shown in Figure 1. In addition to these biomarkers, IL-8, IL-10, and GM CSF presented low positive correlations, with *r* values ranging between 0.30 and 0.50.

Our results suggest that there are a number of biomarkers that appear to be positively associated in varying degrees with the various subjective scores. The most common cytokine was sCD40L, positively associated to all scores except for the NYHA Functional Classification score. Another interesting finding is the relationship of GM-CSF to a wide variety of subjective scores. This cytokine had significant positive association to all scales except for the modified Rankin score. Finally, both VEGF and TNF-alpha were correlated with 3 of the 5 subjective scores, with VEGF not having a significant relation to NHYA and MRC Dyspnea, while TNF-alpha not correlating to NYHA and Rankin. These results suggest that many cytokine biomarkers possess for both the before and after treatment groups positive levels of statistically significant relationship.

### Machine learning classifier validates the labeling of individuals in the dataset group as PASC using cytokine profiles

The individuals in the dataset (data points for the before and after treatment condition) were classified as belonging to the PASC class. This labeling or classification was achieved using the random forest classifier previously published (8) and described in brief in the corresponding methods section. The 36 datapoints of the dataset (18 individuals for each treatment) were labeled with the numeric value of 3, which corresponded to PASC (0 = Normal, 1 = Mild-Moderate, 2 = Severe, and 3 = PASC). Classification off individuals using the random forest model is based on their cytokine profiles. The subjective scores were dropped as the model did not account for these. In addition, because the model was constructed using the 4 states (Normal/Unaffected, Mild-Moderate, Severe and PASC), datapoints can only be classified within one of these states.

Although we observed differences between the before and after treatment conditions (as indicated by the significance of our statistical analysis), with the one tailed-test indicating that the magnitudes in the before condition are greater than after, these changes are not sufficient to lead to a difference in classification using the machine learning model. Although the before and after groups are different when undertaking a statistical comparison, the magnitude in the shift between the groups could not be large enough to be captured as change in state, reflected by the statistical comparison and the subjective scores. Additionally, it is possible that individuals in the after group presented cytokines patterns similar top PASC at the time of sample collection, but if we were to analyze the progression of these samples in a defined time period, we could potentially observe a change in classification, as cytokine profiles continue to change.

## Discussion

The discovery of CD16+ monocytes containing persistent S1 proteins from PASC patients may help further understand its pathophysiology and identify targets for therapy (4). Both CD16+ monocytes subsets, intermediate (CD14+ CD16+) and non-classical (CD14− CD16+), respectively, are known to interact significantly with the endothelium and platelets *via* the fractalkine pathway (24). This suggests that the pathophysiology of PASC may lie with the monocytic-endothelial-platelet axis. Fractalkine, which mediates cell adhesion and leucocyte recruitment, is a transmembrane protein expressed in the brain, colon, heart, and lung, along with endothelial cells and astrocytes. Intermediate monocytes express high levels of both CCR5 and fractalkine receptors, whereas non-classical monocytes express high levels of fractalkine receptors (6, 7). This interaction between fractalkine and fractalkine receptors have been involved in the pathogenesis of atherosclerosis, vasculitis, vasculopathies, and inflammatory brain disorders (5) and could also be contributing to vascular endothelialitis in PASC. Vascular endothelialitis leads to collagen exposure along with platelet activation and adherence *via* glycoprotein 1b-IX-V-receptor (GPIb-IX-V) with collagen-bound von Willebrand factor (vWF) (25). Activated platelets release soluble CD40 ligand (sCD40L) to recruit both neutrophils and monocytes to the vascular lesions (26), thus activating the coagulation cascade. Stimulated platelets also release RANTES which binds to endothelial cells and encourages monocyte adhesion to inflamed endothelial tissues (27) and acts as a chemotactic agent for inflammatory cells. Activated platelets and endothelial cells can also secrete VEGF which induces angiogenesis and microvascular hyperpermeability. VEGF is a diagnostic marker for vasculitic neuropathy and also contributes to a pro-inflammatory-prothrombotic environment (28). While the vascular effects of statins have been well-documented (29), the protective role of maraviroc on the endothelium has also been similarly published (30). Hence, we targeted CCR5 and fractalkine receptors using maraviroc and pravastatin, respectively, hypothesizing that this combination could be therapeutically effective in treating vascular endothelialitis and resolving symptoms associated with PASC.

Neurological symptoms associated with PASC include severe headaches and cognitive impairment (brain fog), along with neuropathy and weakness, necessitating the need for assistance in performing daily tasks. CD14+ CD16+ monocytes are known to transmigrate across the blood brain barrier and play an important role in central nervous system (CNS) immune surveillance. These monocytes were implicated as HIV reservoirs in the CNS causing neuroinflammation, neuronal damage, and cognitive defects (31). We hypothesize that the S1 protein containing CD14+ CD16+ monocytes in PASC patients are also crossing the blood brain barrier and triggering neuroinflammation and inducing neurological symptoms. Both maraviroc and statins are known to cross the blood-brain-barrier, and more specifically, maraviroc has been suggested as treatment for Parkinson’s, neurocognitive impairment, and strokes (32). Interestingly, after the introduction of maraviroc and pravastatin, participants showed a decrease in modified Rankin scale scores (Figure 3) and reported improvement in neurological function and ability. These findings were correlated with a statistically significant decrease in VEGF (*r* = 0.4, *p* = 0.02) and sCD40L (*r* = 0.42, *p* = 0.01) (Table 6), suggesting treatment targeting cytokines associated with vascular endothelialitis correlated with improvement in neurological symptoms.

Autonomic dysfunction such as postural orthostatic tachycardia syndrome (POTS) and light sensitivity has also been associated with PASC. POTS is a syndrome consisting of unexplained tachycardia, dizziness, light-headedness, fainting, and abdominal pain. While the true etiology of POTS has yet to be defined, endothelial dysfunction has been suggested as the pathophysiology (33). There is also evidence that POTS maybe be associated with G-protein-coupled receptor autoantibodies (34). Interestingly, since CCR5 and fractalkine receptor are also G-protein-coupled receptors (9, 35), it is possible that antagonism of these receptors could also inhibit the autonomic effects of these autoantibodies. We observed a statistically significant decrease in COMPASS-31 (Figure 3) scores correlating with statistically significant decreases in VEGF (*r* = 0.6, *p* = 0.0005), sCD40L (*r* = 0.6, *p* = 0.0001), and TNF-alpha (*r* = 0.5, *p* = 0.0026), suggesting that pro-inflammatory macrophage activation may be triggering vascular endothelialitis. Interestingly, elevations in sCD40L have also been associated with sympathoadrenal activation and targeting these vascular markers may address PASC associated dysautonomia (36).

Cardiorespiratory complaints leading to exertional intolerance and limitation of physical activity are very commonly reported by PASC patients. Many PASC patients with cardiac and pulmonary symptoms have undergone extensive workup (EKG, echocardiogram, stress test, pulmonary function testing, etc.) which has not detected any abnormalities or pathologies. Subsequently, current clinical approaches have only been used to treat symptoms with antiarrhythmics, bronchodilators or alpha-adrenergics, instead of addressing the underlying pathophysiology. We observed an improvement in exertional tolerance and physical activity as evidenced by a decrease in NYHA functional classification (Figure 3). This improvement was associated with statistically significant decreases in IL-8 (*r* = 0.4, *p* = 0.03) and GM-CSF (*r* = 0.4, *p* = 0.01). Interestingly, endothelial cells are main producers of IL-8 (37) and statins are known to decrease IL-8 (38). Additionally, maraviroc has been suggested as reducing the cardiovascular risk for acute coronary disease by protecting the endothelium from pro-inflammatory macrophage infiltration (39). These mechanisms potentially support their use in addressing PASC associated cardiac symptoms. We also observed improvement in respiratory symptoms after initiating maraviroc and pravastatin therapy. Participants reported improvements as reflected by a statistically significant decrease in the MRC Dyspnea scale (Figure 3). These responses and improvements correlated with statistically significant decreases in IL-2 (*r* = 0.4, *p* = 0.05), GM-CSF (*r* = 0.6, *p* = 0.0002), sCD40L (*r* = 0.4, *p* = 0.04), and TNF-alpha (*r* = 0.4, *p* = 0.01). Intriguingly, CD16+ monocytes are known to produce large quantities of TNF-alpha and could be activated by the retained S1 proteins (40), causing vascular endothelialitis *via* the fractalkine-fractalkine receptor interaction in pulmonary vasculature. Elevations in sCD40L have been associated with pulmonary arterial hypertension (PAH) (41), while IL-2 can induce pulmonary microvasculature injury and generate an asthma-like bronchoconstriction (42). We previously published a multi-class model score that described an increase IL-2 as a characteristic specific to PASC (8), thus confirming the clinical significance of IL-2 in PASC. Both maraviroc and statins can decrease IL-2 and TNF-alpha (38, 43), which may explain the observed improvements in PASC associated respiratory symptoms. The patient Fatigue Severity Score (FSS) also significantly decreased (Figure 3) after maraviroc and pravastatin which correlated with decrease in sCD40L (*r* = 0.5, *p* = 0.001), VEGF (*r* = 0.5, *p* = 0.001), TNF-alpha (*r* = 0.7, *p* = 4e-5), IL-2 (*r* = 0.6, *p* = 0.0005), and GM-CSF (*r* = 0.5, *p* = 0.004), again suggesting that targeting the monocytic-platelet-endothelial axis can alleviate PASC associated fatigue.

Despite a black box warning for hepatoxicity, maraviroc has demonstrated a strong safety profile in adult, pediatric, and neonatal populations (44, 45). Analysis of the MOTIVATE study demonstrated a low incidence of hepatoxicity with maraviroc even after 96 weeks of treatment at the FDA approved dose of 300 mg B.I.D (46). This influenced our decision to treat with this dose. Hepatic safety was monitored in all the participants by measuring and evaluating AST, ALT, and total bilirubin (LFTs) prior to commencing treatment with maraviroc and every 2 weeks while on treatment. None of participants presented here experienced any clinical signs of hepatotoxicity or elevated liver function serologies while on, or after, treatment. Maraviroc is metabolized by CYP3A4, and we chose to avoid any CYP3A4 metabolizing statins to mitigate any potential drug interactions. This approach guided our decision to treat with pravastatin 10 mg PO daily over the other statins since it is metabolized *via* glucuronidation. However, the therapeutic benefits with other statins have also been observed and should be considered.

Due to rising numbers of post-COVID symptoms and lack of studies and approved therapeutics, many patients have been on different therapeutics. This was reflected in the participants of this case series where some were previously on other therapeutics including ivermectin, fluvoxamine, and prednisone. This presents a significant limitation in this case series because what is unknown is whether these patients were already getting better from these medications before commencing maraviroc and pravastatin. After starting maraviroc and pravastatin, some participants saw symptom relief after 6 weeks, while others needed treatment up to 12 weeks before discontinuing medications. We recognize that studies will need to be conducted to understand this variation in length of treatment between participants. We followed up bi-monthly with these 18 patients for up to 12 weeks post-treatment and no one reported that their symptoms worsened or returned during this period.

Other limitations and weakness of the study were no in-person, objective assessments due to the virtual nature of our clinic, the lack of the control arm and the open label nature of the study. Since immune subset assays were not available for this study, in the future, we would also like to study CD4/CD8 ratios and the amount of S1 proteins in CD16+ monocytes before and after treatment. The results we present in this case series do not replace the need for a double-blinded placebo controlled randomized trial to understand drug efficacy. However, we do believe this study sets the framework for our future clinical trial designs to further investigate the efficacy and usefulness of maraviroc and pravastatin to treat PASC.

## Data availability statement

The original contributions presented in this study are included in the article/supplementary material, further inquiries can be directed to the corresponding author.

## Ethics statement

The studies involving human participants were reviewed and approved by the CCTC IRB committee. The CCTC IRB is financially and operationally independent of the CCTC. No one from this IRB has any financial or research stake in IncellDX or CCTC. This IRB was chosen for cost reasons as this study was self-funded with limited resources. The study protocol was approved by this IRB because it was only designed to collect and analyze patient data from independent physician practices who were responsible for prescribing and monitoring the medications. The CCTC did not and does not prescribe or monitor any medications and has been set up only as a data analytics practice.

## Author contributions

RY, EO, and MZ conceptualized the study. RY organized the study. JG-C and RM-R performed the bioinformatics. RY, JG-C, and RM-R wrote the draft of the manuscript. All authors contributed to revising the manuscript and approved the submitted version.

## Footnotes

1
https://www.sralab.org/rehabilitation-measures/fatigue-severity-scale
