# Corrigendum: Case Series: Maraviroc and pravastatin as a therapeutic option to treat long COVID/Post-acute sequalae of COVID (PASC)

**DOI:** 10.3389/fmed.2024.1375607

**Published:** 2024-05-15

**Authors:** Bruce K. Patterson, Ram Yogendra, Jose Guevara-Coto, Rodrigo A. Mora-Rodriguez, Eric Osgood, John Bream, Purvi Parikh, Mark Kreimer, Devon Jeffers, Cedric Rutland, Gary Kaplan, Michael Zgoda

**Affiliations:** ^1^IncellDX Inc., San Carlos, CA, United States; ^2^Department of Anesthesiology, Beth Israel Lahey Health, Burlington, MA, United States; ^3^Centro de Investigación en Cirugía y Cáncer (CICICA), Universidad de Costa Rica, San Jose, Costa Rica; ^4^Lab of Tumor Chemosensitivity, CIET/DC Lab, Faculty of Microbiology, Universidad de Costa Rica, San Jose, Costa Rica; ^5^Department of Medicine, St. Francis Medical Center, Trenton, NJ, United States; ^6^Department of Emergency Medicine, Novant Health Kernersville Medical Center, Kernersville, NC, United States; ^7^Department of Allergy and Immunology, NYU Langone Tisch Hospital, New York, NY, United States; ^8^Department of Emergency Medicine, New York Presbyterian Hospital, Brooklyn, NY, United States; ^9^Department of Anesthesiology, Stamford Hospital, Stamford, CT, United States; ^10^Rutland Medical Group, Newport Beach, CA, United States; ^11^Department of Community and Family Medicine, Georgetown University Medical Center, Washington, DC, United States; ^12^Department of Medicine, Creighton University School of Medicine, Phoenix, AZ, United States

**Keywords:** long COVID, maraviroc, CCR5 antagonist, PASC, statins, fractalkine (CX3CR1)

In the published article there was an error in the Methodology section.

The sentence previously said:

“The records and immunological lab reports from 18 adult PASC patients treated with maraviroc 300 mg per oral twice daily and pravastatin 10 mg per oral daily from our virtual medical clinic were collected and analyzed.

The 18 participants selected for this case series were from a pool of patients who reported symptom improvement while on maraviroc and pravastatin and who fit the inclusion and exclusion criteria we set below.”

The corrected sentence appears below:

“The medical records and immunological lab reports from 18 adult PASC patients treated with maraviroc 300 mg per oral twice daily and pravastatin 10 mg per oral daily by independent private practice physicians and clinics were collected and analyzed. The CCTC is a virtual consultation group that works in collaboration with these physicians to collect and analyze immunological data. The 18 patients selected for this case series were from a pool of patients who reported symptom improvement while on maraviroc and pravastatin and who fit the inclusion and exclusion criteria we set below:

5 patients were previously treated with ivermectin, 2 with fluvoxamine, and 1 with prednisone.”

In the original manuscript there was an error in the Results section, the Y axis in Figures 2, 3 lacked metric units and the data plots were too clustered for visibility.

In Figure 2, we created absolute difference for before and after cytokine measurements for each of the 14 cytokines since the original graph was too clustered and hard to interpret. In the corrected version, we also added the y-axis legend (in pg/ml) for clarity.

**Figure 2:**
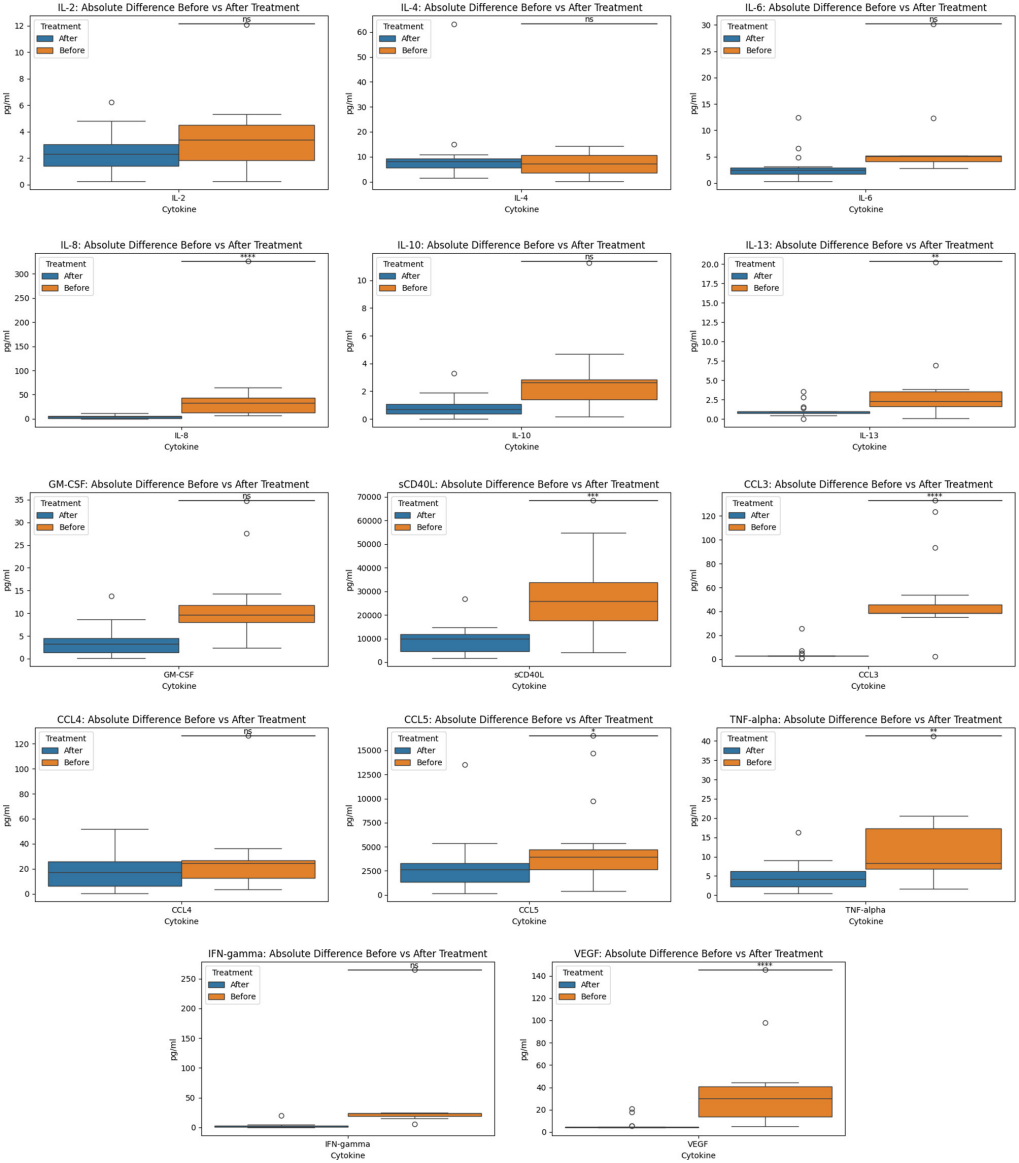
Before and after treatment individual cytokine measurement comparisons. The box plot represent the statistical comparison using the Wilcoxon paired test between the two treatment groups (before and after).

In Figure 3, we added box plots and added the y-axis legend for clarity. The following revised Figures are below. We renamed the Y-axis to “Subjective Score Values” and the X-axis to “Subjective Scores.”

**Figure 3:**
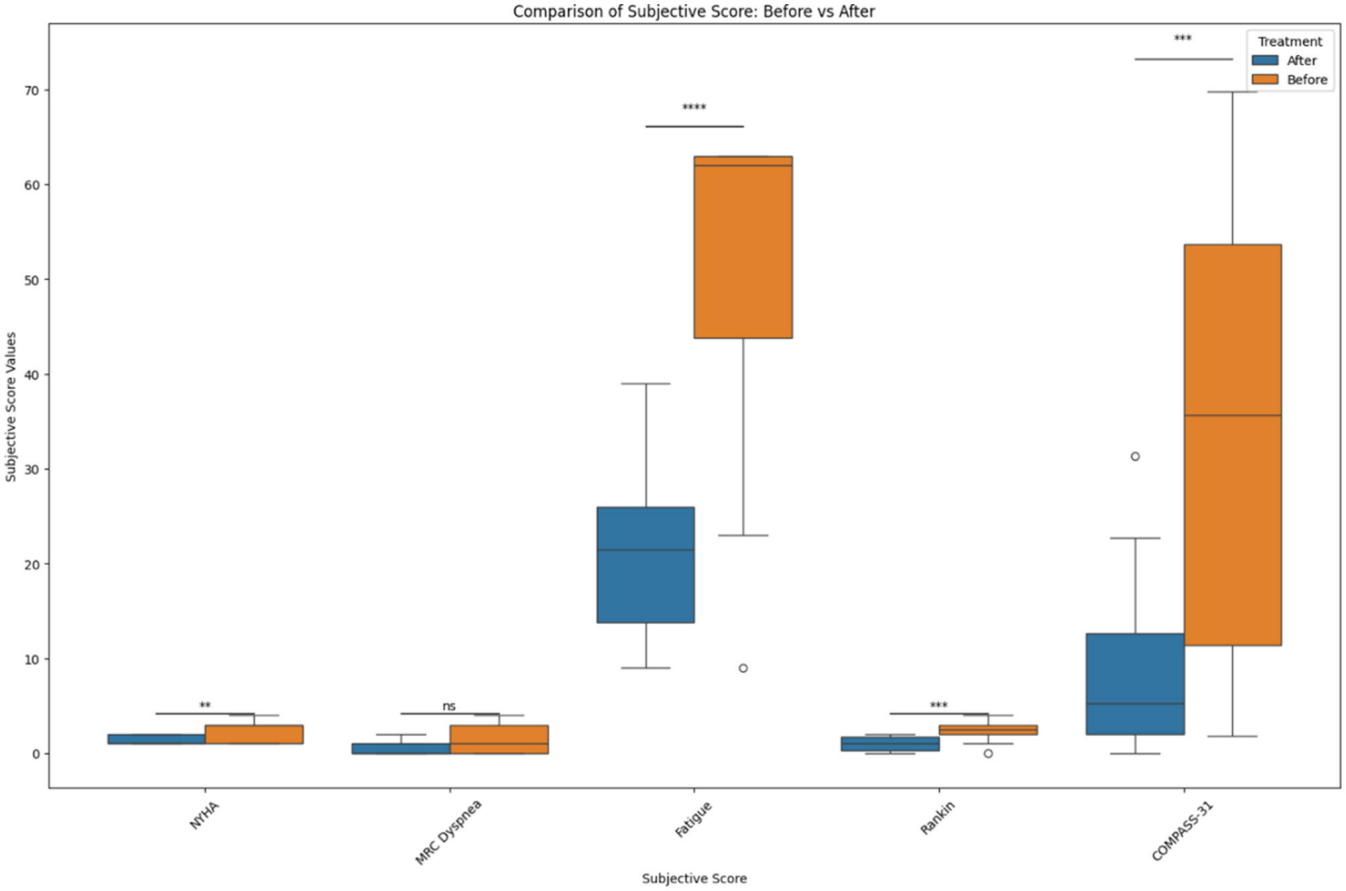
Before and after treatment individual subjective score comparisons. The box plot represents the statistical comparison using the Wilcoxon paired test between the two treatment groups (before and after). Statistical significance intervals are represented with asterisks (^*^), where ns indicates non-significant. ^*^1.00e-02 < *p* ≤ 5.00e-02, ^**^1.00e-03 < *p* ≤ 1.00e-02, ^***^1.00e-04 < *p* ≤ 1.00e-03, and ^****^p ≤ 1.00e-04.

In the published article, there was an error in the Ethics statement. The sentence previously stated:

“The studies involving human participants were reviewed and approved by CCTC Ethics and IRB Committee. The patients/participants provided their written informed consent to participate in this study.”

The corrected sentence appears below:

## Ethics statement

The studies involving human participants were reviewed and approved by the CCTC IRB committee. The CCTC IRB is financially and operationally independent of the CCTC. No one from this IRB has any financial or research stake in IncellDX or CCTC. This IRB was chosen for cost reasons as this study was self-funded with limited resources. The study protocol was approved by this IRB because it was only designed to collect and analyze patient data from independent physician practices who were responsible for prescribing and monitoring the medications. The CCTC did not and does not prescribe or monitor any medications and has been set up only as a data analytics practice.

In the published article, there was an error in the Funding statement. We did not state that there was no funding received for the study. The correct Funding statement appears below.

